# Inwardly Rectifying Potassium Channel Kir2.1 and its “Kir-ious” Regulation by Protein Trafficking and Roles in Development and Disease

**DOI:** 10.3389/fcell.2021.796136

**Published:** 2022-02-09

**Authors:** Natalie A. Hager, Ceara K. McAtee, Mitchell A. Lesko, Allyson F. O’Donnell

**Affiliations:** Department of Biological Sciences, University of Pittsburgh, Pittsburgh, PA, United States

**Keywords:** KCNJ, development, protein trafficking, Kir, QT syndrome, Andersen-Tawil syndrome, KCNJ2 and IRK1

## Abstract

Potassium (K^+^) homeostasis is tightly regulated for optimal cell and organismal health. Failure to control potassium balance results in disease, including cardiac arrythmias and developmental disorders. A family of inwardly rectifying potassium (Kir) channels helps cells maintain K^+^ levels. Encoded by *KCNJ* genes, Kir channels are comprised of a tetramer of Kir subunits, each of which contains two-transmembrane domains. The assembled Kir channel generates an ion selectivity filter for K^+^ at the monomer interface, which allows for K^+^ transit. Kir channels are found in many cell types and influence K^+^ homeostasis across the organism, impacting muscle, nerve and immune function. Kir2.1 is one of the best studied family members with well-defined roles in regulating heart rhythm, muscle contraction and bone development. Due to their expansive roles, it is not surprising that Kir mutations lead to disease, including cardiomyopathies, and neurological and metabolic disorders. Kir malfunction is linked to developmental defects, including underdeveloped skeletal systems and cerebellar abnormalities. Mutations in Kir2.1 cause the periodic paralysis, cardiac arrythmia, and developmental deficits associated with Andersen-Tawil Syndrome. Here we review the roles of Kir family member Kir2.1 in maintaining K^+^ balance with a specific focus on our understanding of Kir2.1 channel trafficking and emerging roles in development and disease. We provide a synopsis of the vital work focused on understanding the trafficking of Kir2.1 and its role in development.

## 1 Introduction

### 1.1 *KCNJ* Family

**FIGURE 1:**
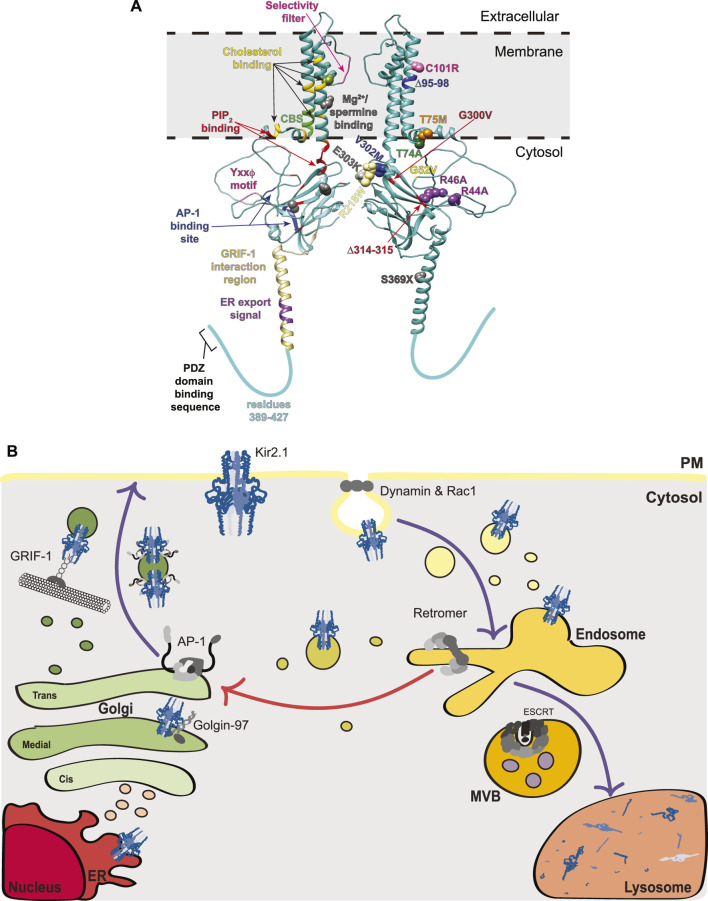
Structural mapping and trafficking model of Kir2.1. **(A)** A model for the Kir2.1 monomer was generated using AlphaFold2 (accession #P63252) (Jumper et al., 2021). Interestingly, the AlphaFold2 predicted structure for Kir2.1 reveals an extended alpha-helix spanning residues 358–388 that is not observed in the crystal structures for Kir channels as this region was removed from the protein prior to crystallization. For simplicity, a dimer of Kir2.1 subunits is shown, rather than the tetramer that would typically exist at the PM. The left subunit on our dimer denotes binding motifs for trafficking factors and the right subunit highlights disease-causing mutations associated with aberrant Kir2.1 trafficking. Left: Selectivity filter (pink), Mg^2+^/spermine binding site (gray and green where it overlaps with a cholesterol binding residue), cholesterol binding residues (yellow except where they overlap with a Mg^2+^/spermine binding site [light green], a Cav3 binding site [dark green], or a PIP_2_ binding site [orange]), Cav3 binding site (CBS, bright green and one dark green where it overlaps with a cholesterol binding site), PIP_2_ binding residues (red and one orange where it overlaps with a cholesterol binding site), Yxxϕ motif (pink), AP-1 binding site (blue), GRIF-1 interaction region (tan), and ER export signal (purple). Right: The side chains of residues mutated in disease and linked to defective protein trafficking are shown as spheres. Text indicating the specific mutation associated with each is color-coordinated. **(B)** Trafficking model of Kir2.1 highlighting interacting components at each stage including: Golgin-97, AP-1, GRIF-1, dynamin, Rac1, retromer, and ESCRT. Purple arrows indicate well-studied trafficking routes for Kir2.1 whereas the red arrow denotes a putative endosome-Golgi recycling pathway. MVB, multivesicular bodies; ER, endoplasmic reticulum; PM, plasma membrane.

Potassium homeostasis is needed for cell health and cellular potassium (K^+^) balance is tightly regulated. Specifically, K^+^ is essential for normal functioning of nerve and muscle cells due to its contribution to membrane potential (Bia and DeFronzo, 1981; Youn and McDonough, 2009). A family of inwardly-rectifying potassium (Kir) channels plays a central role in this regulation. The Kir channels are encoded by 16 *KCNJ* genes and their function impacts a wide range of cellular processes. Every Kir channel consists of four subunits–either homo or hetero-tetramers–each with two transmembrane domains, cytoplasmic N- and C-termini, and an extracellular loop that forms the pore-lining selectivity filter (Figure 1A) (de Boer et al., 2010; Hibino et al., 2010). Although fundamentally similar in structure, Kir family members diverge in their K^+^ shuttling properties. For example, there are “strong” (Kir2 and Kir3), “intermediate” (Kir4), and “weak” (Kir1 and Kir6) rectifiers. This functional plasticity is beneficial as Kir channels are expressed in diverse cell types, including cardiomyocytes, neurons, blood cells, osteoclasts, endothelial cells, glial cells, epithelial cells, and oocytes, each with distinct requirements for K^+^ (Hibino et al., 2010). Here we focus on the Kir2.x subfamily (Kir2.1-4 and Kir2.6), also known as the “classical” Kir channels.

### 1.2 Overview of Kir2.x Subfamily

Classical Kir channels are comprised of the Kir2 subfamily (referred to hereafter as Kir2.x), which includes Kir2.1 (*KCNJ2*), Kir2.2 (*KCNJ12*), Kir2.3 (*KCNJ4*), Kir2.4 (*KCNJ14*) and Kir2.6 (*KCNJ18*) (Table 1). These channels are constitutively active and have a strong inward K^+^ rectification that is essential in establishing a stable, negative resting membrane potential in excitable cell types like cardiomyocytes (de Boer et al., 2010). The strong inward rectification results from a voltage dependent block of the channel by intracellular polyamines and Mg^2+^. Specifically, these positively charged polyamines and Mg^2+^ are pulled into the channel when the membrane potential is more positive than the equilibrium potential for K^+^, thus sterically and electrostatically blocking K^+^ efflux, as seen in excitable cells (Cheng et al., 2013). This contribution to resting membrane potential aids in delaying new action potential firing and ensures an appropriate QT interval. Due to this imperative role in excitable cells, it is not surprising that defective channels have disastrous results. For example, the well-studied, hypomorphic ∆314–315 mutation (Figure 1A; Table 1) impairs Kir2.1 trafficking to the plasma membrane (PM) resulting in Andersen-Tawil syndrome (ATS) (see Sections 2.1-2.3). ATS is characterized by periodic paralysis, repolarization changes in electrocardiograms, and developmental abnormalities including dysmorphic features, wide-set eyes, low-set ears, broad forehead, small jaw and head, cleft palate, and curved, fused and/or shortened digits (Plaster et al., 2001; Pérez-Riera et al., 2021). Alternatively, hypermorphic mutations, such as E299V, M301K and D172N, cause excess K^+^ flux resulting in short QT syndrome and increased risk of sudden cardiac death (Li et al., 2004; Priori et al., 2005; Ambrosini et al., 2014). Due to the serious ramifications of hypo- or hypermorphic Kir2.1 function, intensive study has revealed many mechanistic details of channel regulation (see Section 2.2).

**TABLE 1:** Summary of Kir2.x disease causing mutations linked to protein trafficking

Channel	Gene	Aliases	Localization	Disease	Trafficking mutations	References
Kir2.1	*KCNJ2*	IRK1 LQT7 IRK-1 HIRK1	brain, eye, heart, smooth muscle, skeletal muscle, placenta, kidney	Andersen-Tawil syndrome (Long QT syndrome)	∆S314-Y315; S369X;	(Lopes et al., 2002; Bendahhou et al., 2003; Ballester et al., 2006; Tani et al., 2007; de Boer et al., 2010; Doi et al., 2011; Ma et al., 2011; Ambrosini et al., 2014; Gélinas et al., 2017)
					∆95–98; V302M^a^; C101R;	
					T75M;	
					G52V;	
					G300V^a^; E300K^a^;	
					T74A^a^;	
					R218W	
				Short QT syndrome	K346T	
				Familial atrium fibrillation	−	
Kir2.2	*KCNJ12*	IRK2 Kir2.2v	brain, eye, heart, smooth muscle, skeletal muscle, kidney	Familial Esophageal Squamous Cell Carcinoma	−	(de Boer et al., 2010; Khalilipour et al., 2018)
		KCNJN1				
Kir2.3	*KCNJ4*	HIRK2	brain, eye, heart, smooth muscle	Parkinson’s disease	−	(Shen et al., 2007; de Boer et al., 2010)
		HRK1				
		IRK3				
		HIR				
Kir2.4	*KCNJ14*	IRK4	brain, eye, heart, smooth muscle	−	−	(de Boer et al., 2010)
Kir2.6	*KCNJ18*	TTPP2	skeletal muscle	Thyrotoxic hypokalemic periodic paralysis	R399X^a^;	(de Boer et al., 2010; Ryan et al., 2010; Cheng et al., 2011)
					Q407X^a^;	
					R43C;	
					A200P	

a Denotes mutations that are suspected to impair protein trafficking of Kir2.x but are not yet fully defined.

The degree of K^+^ rectification by Kir channels is determined by the charge of key residues in the second transmembrane helix (TM2). A negatively charged residue (D172 in Kir2.1) leads to strong inward K^+^-rectification due to increased affinity for intracellular polyamines/Mg^2+^, whereas an uncharged residue (N171 in Kir1.1 or D172N mutant of Kir2.1), causes a weak inward K^+^ rectification due to diminished binding to pore-blocking polyamines/Mg^2+^ (Lu and MacKinnon, 1994; Stanfield et al., 1994; Wible et al., 1994; Yang et al., 1995). Kir2.x family members can form functional homo- and hetero-tetramers (Tinker et al., 1996; Preisig-Muller et al., 2002) and are expressed in a variety of cell types including brain, eye, heart, smooth and skeletal muscle, and kidney. Select Kir members are expressed during embryonic development in the heart (Kir2.1–2.4), brain (Kir2.1-Kir2.3), limbs (Kir2.1), ear (Kir2.1), epithelia (Kir2.2), metanephros (Kir2.2), and peripheral nervous system (Kir2.2) (Karschin and Karschin, 1997; Grandy et al., 2007; Harrell et al., 2007; Ruan et al., 2008). Our understanding of the role of Kir2.x family members in development is discussed in Section 3.

We have considerable knowledge on Kir channel function, yet there remain critical facets of Kir physiology to be discovered. For example, we have yet to fully elucidate the contributions of Kir channels to development, which requires rapid alterations in membrane potential; defects in Kir function during development cause many developmental disorders. In addition, while great strides have been made in understanding Kir protein trafficking and stability at the PM, there remain unidentified molecular players ushering these channels to and from the cell surface. In this review, we provide a synopsis of Kir2.x family members, focusing on Kir2.1 trafficking pathways and roles in embryonic development. We emphasize how Kir2.x regulatory pathways are disrupted to give rise to disease states.

## 2 Regulation of Kir2.x by Protein Trafficking and its Role in Disease

### 2.1 Mapping the Kir2.1 Trafficking Itinerary: Machinery and Motifs

All Kir2.x subfamily members have an endoplasmic reticulum (ER) export sequence in their cytoplasmic C-termini (consensus sequence FxYENEV; ^374^FCYENEV^380^ for Kir2.1), which is essential for Kir2.x ER exit (Ma et al., 2001; Stockklausner et al., 2001) (Figures 1A,B). In the Golgi, Golgin 97—which belongs to the Golgin family of membrane and cytoskeleton tethers that capture transport vesicles for fusion to Golgi compartments—binds directly to the cytoplasmic C-terminal tail of Kir2.1 *via* its GRIP (golgin-97, RanBP2alpha, Imh1p and p230/golgin-245) domain to help ensure that Kir2.1-containing vesicles reach their destination in the *trans*-Golgi network (TGN) (Taneja et al., 2018) (Figure 1B). Knockdown of Golgin-97 in COS-7 cells causes Kir2.1 accumulation in early Golgi compartments and prevents it from reaching the TGN (Taneja et al., 2018).

Upon reaching the TGN, Kir2.1 interacts directly with adaptin protein complex 1 (AP-1), which is a cargo selective adaptor for Golgi- and endosome-derived, clathrin-coated transport vesicles (Bonifacino and Traub, 2003) (Figure 1B). Prior association with Golgin-97 is posited to help Kir2.1 incorporation into AP-1 associated vesicles (Taneja et al., 2018). AP-1 is a key mediator of Kir2.1 trafficking to the PM and the disease causing ATS mutant, Kir2.1-∆314–315, fails to exit the Golgi due to deficient AP-1 binding (Ma et al., 2011). The Kir2.1-AP-1 interaction interface is complex and requires N- and C-terminal residues of Kir2.1. Earlier studies identified key basic residues in the N-terminus (R44 and R46 in Kir2.1) that when mutated to alanine block Golgi export (Figure 1A) (Stockklausner and Klöcker, 2003). These same N-terminal residues form an interface with C-terminal residues (Y315, E319, I320 and W322) that permit AP-1 binding and Kir2.1 Golgi export. This Kir2.1-AP-1 interaction motif is structurally distinct from conventional AP-1 motifs (reviewed in (Traub and Bonifacino, 2013)), yet is conserved in many Kir proteins (Li et al., 2016); this represents an exciting new way for membrane proteins to engage the AP-1 trafficking machinery. It should be noted that earlier studies identified a more ‘classical’ AP-1 binding sequence in the C-terminus of Kir2.1 with the consensus YxxΦ, where x is any amino acid and Φ is a bulky hydrophobic amino acid (corresponds to amino acids ^242^YIPL^245^ in Kir2.1; Figure 1A), as important for Golgi-to-PM trafficking (Hofherr et al., 2005). However, more recent mutational analysis makes it unclear if this patch is important for Kir2.x family member’s sorting to the surface (Li et al., 2016). Many Kir family members that do not transit to the PM possess the conserved YxxΦ motif at this location, so if this sequence is involved the flanking residues must help its function. In addition to AP-1 regulation of its Golgi-to-PM transit, Kir2.1 binds to γ-aminobutyric acid type A (GABA_A_) receptor interacting factor-1 (GRIF-1; aka trafficking kinesin protein 2 [TRAK2]), which links kinesin heavy chains to a specific cargo and may play a role in anterograde trafficking of vesicles and organelles (Smith et al., 2006). The GRIF-1 interaction with Kir2.1 was roughly mapped using yeast 2-hybrid analyses, and lies within the N-terminal region of GRIF-1 and amino acids 348–396 in the C-terminus of Kir2.1, which are predicted to form an extended alpha helix (Figure 1A). The GRIF1-Kir2.1 interaction is thought to promote PM localization of Kir2.1 (Figure 1B) (Grishin et al., 2006).

Once at the PM Kir2.1 controls the entry of K^+^ into cells. Kir2.1 activity, partitioning and dwell time at the PM are influenced by several factors. First, Kir2 channels at the cell surface bind to phosphatidylinositol 4,5-bisphosphate (PIP_2_), which is an important agonist required for channel opening. X-ray crystallography studies demonstrate that PIP_2_ binding changes the angle between the TM and cytosolic domains, with the cytosolic C-terminal domain migrating towards the membrane causing a subsequent rotation of hydrophobic side chains within the central K^+^-transporting pore to permit K^+^ passage (Hansen et al., 2011; Huang et al., 1998). Further, the interaction of Kirs with PIP_2_ has broad ranging consequences on their modulation by other factors, including pH, phosphorylation and protein-protein associations (Logothetis et al., 2007; Du et al., 2004; Rosenhouse-Dantsker et al., 2012). Initially, Kir channel residues that interact with PIP_2_ were identified using functional approaches, including detailed mutagenesis mapping in Kir2.1 and other Kir channels (Logothetis et al., 2007; Du et al., 2004; Lopes et al., 2002). The Kir2.1-PIP_2_ interface was then confirmed by co-crystallization and, like the binding site for AP-1, the PIP_2_ binding site is made of many spatially separated residues that in combination create a binding surface in the folded channel (Figure 1A) (Hansen et al., 2011). PIP_2_ also alters channel conformation to allow proper regulation by Mg^2+^; disruption of PIP_2_ binding results in irreversible inhibition of Kir2.1 by Mg^2+^ (Du et al., 2004). Importantly, mutations in the PIP_2_ binding residues of Kir2.1 are associated with ATS (see Section 2.2 below).

Second, Kir2.1 at the PM partitions primarily to cholesterol- and flotillin-rich membrane fractions (Ambrosini et al., 2014; Tikku et al., 2007). All members of the Kir2.x family are cholesterol sensitive. Increases in membrane cholesterol elevate the probability of channel closure due to cholesterol-responsive sites (Romanenko et al., 2004; Rosenhouse-Dantsker et al., 2011). These cholesterol responsive sites are unique from the putative binding sites for cholesterol in Kir2.1, the latter of which were mapped to the TMD α-helices (residues L69, A70, V77, L85, V93, S95, I166, V167, I175, M183, Y68, C76, I79, F159, and S165) using computational and mutagenesis strategies (Figure 1A) (Rosenhouse-Dantsker et al., 2011; Rosenhouse-Dantsker et al., 2013). The putative cholesterol binding sites overlap partially with an inverted cholesterol recognition amino acid consensus, or CARC, motif [consensus sequence (R/K)X_1-5_(Y/F)X_1-5_(L/V) where X is any amino acid and corresponds to residues R67-F73-V77 in Kir2.1], however a second putative CARC motif (R82-F88-L90) does not appear to bind cholesterol (Rosenhouse-Dantsker et al., 2013). Interestingly, using similar approaches, putative cholesterol binding sites were defined in Kir3.x family members, and some of these have been substantiated by recent cryoEM studies (Bukiya et al., 2017; Bukiya and Rosenhouse-Dantsker, 2017; Mathiharan et al., 2021). While the cholesterol binding sites map to similar regions in the TMDs of Kir2.1 and Kir3.x family members (compared in (Rosenhouse-Dantsker, 2019)), cholesterol inhibits Kir2.1 but activates Kir3.x channels, presenting an interesting functional dichotomy between these two family members. Importantly, while binding to cholesterol and PIP_2_ occurs largely at discreet loci, crosstalk between these regulators has been identified for Kir2.1 and Kir3.4 (Bukiya and Rosenhouse-Dantsker, 2017; Rosenhouse-Dantsker et al., 2014). For Kir2.1 and Kir2.3, reduced cholesterol in the membrane increases the Kir2.x-PIP_2_ interaction (Rosenhouse-Dantsker et al., 2014), although this does not fully explain the cholesterol responsiveness of these channels. In contrast, for Kir3.4 cholesterol and PIP_2_ act in concert to increase channel function (Bukiya and Rosenhouse-Dantsker, 2017), demonstrating that the interplay between PIP_2_ and cholesterol is channel dependent and the structural alterations upon cholesterol binding must be distinct in these two subfamilies. In addition to these considerations, cholesterol/flotillin-rich membranes are associated with caveolins, which are TM proteins that promote membrane curvature during caveolae-mediated internalization. Kir2.1 interacts with caveolins Cav1, Cav2, and Cav3 (Ambrosini et al., 2014; Tikku et al., 2007; Han et al., 2014; Vaidyanathan et al., 2018; Ikezu et al., 1998). Caveolae, or little ‘caves’ within the PM, are associated with elevated cholesterol and control the endocytosis of many membrane proteins, including several K^+^ channels. Although Cav1 is a negative regulator of Kir2.1 and Cav3 mutations can themselves cause long-QT syndrome, perhaps due to reduced Kir function, the role of caveolae in Kir2.x endocytosis remains largely unexplored (Han et al., 2014; Vaidyanathan et al., 2018). The binding site for Cav3 in Kir2.1 resides between amino acids 81–88 in the N-terminus of this protein, where a canonical ΩxΩxxxxΩ Cav3-binding motif is located (Ω is any aromatic amino acid; ^81^WRWMLVIF^88^ for Kir2.1 and shown as CBS in Figure 1A) (Vaidyanathan et al., 2018).

Third, Kir2.x channels form macromolecular complexes at the PM which help stabilize them at the cell surface. For example, Kir2.x at the cell surface interacts with many postsynaptic density, discs large, and zonaula occludens (PDZ)-domain containing proteins, as many Kir channels have a class I PDZ domain-recognition sequence (^440^SEI^442^ for Kir2.1) in their C-termini. These interactions play an important role in Kir2 localization and partitioning at the membrane (Cohen et al., 1996; Songyang et al., 1997). Kir2.x channels interact with the PDZ domain-containing proteins in the MAGUK (membrane-associated guanylate kinase) family, including SAP97, PSD-95, Chapsyn 110, and CASK, which are molecular scaffolding proteins that regulate signaling and trafficking of many receptors and ion channels (Cohen et al., 1996; Leonoudakis et al., 2001; Sheng and Sala, 2001; Leonoudakis et al., 2004a; Leonoudakis et al., 2004b). In the brain, Kir2.x interacts with several scaffolding and structural proteins, including SAP97, CASK, Veli, Mint and actin-binding LIM proteins, that are important for channel surface retention (Leonoudakis et al., 2004b). In addition, Kir2.1 co-expression with Na_v_1.5, a voltage-gated sodium channel found in the heart, reduces Kir2.1 internalization by forming a macromolecular complex with PDZ-domain-containing SAP97 (Milstein et al., 2012). A more detailed review of Kir2.1 functions within macromolecular complexes is found in Willis et al. (Willis et al., 2015).

Finally, recent exciting work examined the role of focal adhesions (FAs) in Kir2.1 PM localization, where confocal microscopy identified Kir2.1 accumulating in PM subdomains proximal to FAs. The larger the FA area the higher the amplitude of Kir2.1 current, suggesting that FAs promote Kir2.1 PM accumulation; indeed Kir2.1 at areas distal to FAs is more thoroughly endocytosed (Sengupta et al., 2019). This phenomenon is likely due to local inhibition of dynamin-dependent endocytosis at FAs (Sengupta et al., 2019). Thus, a bevy of molecular factors regulates Kir2.1 and other Kir2.x family members trafficking and partitioning at the PM to promote optimal function of these critical K^+^ channels.

Kir2.1 surface abundance must be controlled by regulated endocytosis and subsequent lysosomal degradation, as has been described for many channels and transporters (Estadella et al., 2020). Internalization from the PM typically proceeds via clathrin-mediated or clathrin-independent endocytosis (CME and CIE, respectively) (von Zastrow and Sorkin, 2021). Unlike other Kir family members’, there are currently very few descriptions of Kir2.1 endocytic regulation; for Kir2.3, Kir6.2 and Kir1.1(ROMK) there is clear evidence for CME (Zeng et al., 2002; Mankouri et al., 2006; Mason et al., 2008; Ortega et al., 2012). Interestingly, for Kir2.3 a non-canonical adaptin-protein complex 2 (AP-2) binding site with a recognition motif of ΦΦxΦΦ spans residues ^412^IIRML^416^ (Ortega et al., 2012). Since AP-2 is an adaptor that recruits clathrin to endocytic sites, this clearly defines a role for CME in Kir2.x family trafficking. However, this site is not conserved in Kir2.1, despite the >60% conservation between Kir2.1 and Kir2.3. In addition to this non-canonical AP-2 binding site in Kirs, a canonical AP-2 binding site exists in Kir6.2 (^330^YSKF^333^ which is consistent with a YxxΦ AP-2 recognition motif) (Mankouri et al., 2006). This motif is nearly perfectly conserved in Kir2.1 (^341^YSRF^344^), but its ability to bind AP-2 is yet to be examined. This YxxΦ motif is distinct from the one between amino acids 242–245 that was initially considered as important for Golgi exit (as discussed above). Interestingly, an earlier *Xenopus* oocyte study suggests Kir2.1 is regulated by CME. Specifically, caging of Y242 of Kir2.1 resulted in increased Kir2.1 PM localization; when Y242 was uncaged, Kir2.1 endocytosis increased, suggesting that phosphorylation of Kir2.1 at this site may be important for internalization (Tong et al., 2001). Further, a dominant-negative mutant of dynamin prevented the Y242-induced internalization of Kir2.1, suggestive of a role for CME. However, both CME and CIE—including caveolin and Rho-dependent internalization mechanisms—can engage dynamin, a small GTPase that drives membrane scission events (Mayor et al., 2014; Sandvig et al., 2018). Thus, the fact that dynamin is involved does not preclude CIE from regulating Kir2.1. More recent studies have looked at the role of the Rho-family GTPases (including Rho, Rac and Cdc42), which have broad cellular functions including regulating protein trafficking, cytoskeletal dynamics, and cell-cell adhesion, in controlling Kir2.1 (Boyer et al., 2009; Mayor et al., 2014; Hodge and Ridley, 2016). Using pharmacological inhibition or dominant negative mutations that impede the Rho, Rac and Cdc42 GTPases, the authors demonstrate that impaired Rac1 increases Kir2.1 at the PM, as evidenced by increased conductance measures and microscopy (Boyer et al., 2009). The impact of Rac1 is lost when cells express a dynamin mutant that prevents vesicle scission, suggesting that the role of Rac1 is tied to Kir2.1 endocytosis. Rac1, and other Rho-family GTPases, can contribute to clathrin-independent, dynamin-dependent endocytic pathways and perhaps this is how Rac1 regulates Kir2.1 (Grassart et al., 2008). Interestingly, related Kir2.x family members were not regulated by Rac1, as Rac1 mutants did not alter their distribution. While Kir2.1 internalization from the PM must be regulated, it remains unclear which endocytic pathway and sorting factors control this. It is tempting to speculate that Kir2.1 uses a clathrin-independent route given its regulation by the Rac1 GTPase as well as its propensity to cluster in cholesterol-rich membrane domains and interact with caveolins, but this remains to be explored.

While the endocytic route for Kir2.1 remains enigmatic, this channel transits to the lysosome for degradation as lysosomal inhibitors, such as NH_4_Cl, chloroquine, and leupeptin, increase Kir2.1 protein levels (Jansen et al., 2008). Further studies in yeast (as described below) identified the endosomal sorting complex required for transport (ESCRT) machinery as important for controlling Kir2.1 function at the PM. The involvement of ESCRTs was confirmed by studies in HeLa cells where knockdown of ESCRT components increased Kir2.1 abundance (Kolb et al., 2014).

### 2.2 Disease-Linked Trafficking Mutations in Kir2.1

Identifying mutations causing Kir2.x loss-of-function, many of which are associated with ATS, has advanced our understanding of Kir2.x trafficking. A good example of this is the Kir2.1-∆314–15 mutation, which, as indicated earlier, disrupts an unconventional AP-1 binding site in Kir2.1 and prevents cell surface expression (Figures 1A,B; Table 1) (Bonifacino and Traub, 2003; Ma et al., 2011). Confocal microscopy reveals retention of ∆314–315 in the Golgi and pulse-chase experiments show that these channels never make it to the PM. Recent BioID proximity proteomics analyses of Kir2.1-∆314–315 compared to wild-type Kir2.1 further validated the spatial distribution differences between these two channels; factors copurifying with Kir2.1-∆314–315 were enriched for proteins involved in intracellular trafficking and transport (Park et al., 2020). Thus Kir2.1 must interact with, and likely be incorporated into, AP-1 and clathrin-coated vesicles at the TGN which then transit to the PM (Figure 1B). Patients with the Kir2.1-∆314–315 mutation have severe ATS phenotypes (Plaster et al., 2001).

Mutations in regions needed for effective trafficking of Kir2.1 result in varying degrees of ATS. For example, patients with the S369X mutation, encoding a premature stop codon and truncation of Kir2.1 C-terminus, lack the ER export motif (^374^FCYENE^389^). This dramatically reduces Kir2.1 at the PM and lowers channel activity to cause severe disease (Doi et al., 2011). However, in a Kir2.1 tetramer containing both wild-type and S369X Kir2.1 subunits the intact ER-export signal from the wild-type subunits allows for improved ER-export and increased Kir2.1 function. Patients heterozygous for S369X therefore display only mild ATS symptoms (Doi et al., 2011). While some Kir2.1 mutations can be compensated for in *trans* by assembly with wild-type Kir2.1 subunits, like the S369X mutation, others act as dominant-negatives impairing the function of co-assembled wild-type Kir2.1 subunits. For example, Kir2.1-∆314–315 assembly with wild-type Kir2.1 subunits still fails to traffic to the PM, presumably because the association with AP-1 is not stable enough (Bendahhou et al., 2003).

There also exist ATS-linked mutations that affect the ability of Kir2.1 to reach the PM, though for many of these the defect seems likely linked to protein folding or insertion into the ER rather than subsequent protein trafficking. These mutations include a deletion in the first membrane span (∆95–98) that is thought to interfere with Kir2.1 membrane insertion and results in cytoplasmic channel localization (Table 1) (Bendahhou et al., 2003). Mutation of V302M, which lies at the end of one of the beta-sheets, and C101R, in the first TM helix, each reduce Kir2.1 PM localization and result in cytosolic localizations, also suggestive of misfolding (Figure 1A; Table 1) (Bendahhou et al., 2003; Ballester et al., 2006).

Kir2.1-T75M and -T74A each mutate residues needed for PIP_2_ binding. T74A exhibits wild-type trafficking but altered activity due to diminished PIP_2_ association (Ballester et al., 2006). In contrast, T75M has less PM localized channel than is associated with wild-type Kir2.1. Trafficking of the T75M channel to the PM is restored in *trans* by co-expression with wild-type Kir2.1, however, these hetero-tetrameric complexes display diminished K^+^ shuttling, potentially due to defective PIP_2_ regulation and a subsequent increase in Mg^2+^ channel blocking (Tani et al., 2007). Other disease-causing mutants (R218W, G300V, E303K) also disrupt PIP_2_ binding but are not yet reported to disrupt Kir2.1 trafficking (Lopes et al., 2002). The Kir2.1-G52V mutation, which is adjacent to the PIP_2_ binding residue H53, causes Golgi retention of the channel. The G52V mutation may cause a conformational change in the channel that disrupts the nearby AP-1 binding site (residues 44–46 and 314–322), and this could explain Golgi-retention. Alternatively, the G52V mutation may act in a similar fashion to the T75M mutant by altering PIP_2_ interaction and thereby disrupting trafficking (Gélinas et al., 2017).

Finally, the only gain-of-function, trafficking-defective mutant identified to date is Kir2.1-K346T, which is linked to short QT syndrome 3 (Ambrosini et al., 2014). This protein is retained at the PM, has diminished interaction with caveolins and increases partitioning of the channel to cholesterol-poor membranes. Furthermore, ubiquitination of the K346T mutant is reduced (Ambrosini et al., 2014), which may indicate that this is a site for ubiquitination that stimulates endocytosis, although this remains to be experimentally explored.

### 2.3 Kir2.1 Trafficking Studies in *Saccharomyces cerevisiae*

Studies in *Saccharomyces cerevisiae* as a heterologous expression system for Kir2.1 have contributed meaningfully to our knowledge of Kir2.1 trafficking and degradation (Kolb et al., 2014; Hager et al., 2018). Kir2.1 is ectopically expressed in yeast mutants lacking endogenous potassium channels, Trk1 and Trk2. In this model, Kir2.1 is the primary importer for K^+^ into yeast cells, so when these cells are grown in K^+^ restrictive medium, cells are dependent on Kir2.1; growth on K^+^-limiting medium serves as a proxy for Kir2.1 function at the PM. Leveraging this system, Kolb *et al.* performed a genome wide screen to identify gene deletions that improve Kir2.1-dependent growth on low K^+^ medium in yeast. Over 60% of the top candidates identified were trafficking factors including retromer (retrieval of vacuolar-targeted proteins), AP-1, and ESCRT (Kolb et al., 2014). This screen demonstrated that in yeast: 1) Kir2.1 is an ER-associated degradation (ERAD) substrate requiring Cdc48, an AAA-ATPase, Hrd1 and Doa10, the ER-associated E3 ubiquitin ligases, and Ssa1, a cytoplasmic Hsp70 chaperone, for its retrotranslocation and degradation (Kolb et al., 2014), and 2) deletion of several ESCRT pathway components (Did2, Vps36, Vps27, Vps22, Vps2, Vps23, Vps37, Mvb12, and Vta1) increase Kir2.1-dependent growth on low K^+^ medium, suggesting that ESCRT is needed to degrade Kir2.1. ESCRT-mediated degradation of Kir2.1 likely occurs via post-endocytic trafficking of the channel to the vacuole, which is the yeast equivalent of the lysosome. Excitingly, the lysosomal dependence on ESCRT-mediated degradation of Kir2.1 was validated in HeLa cells, demonstrating that the yeast system defines relevant regulators of Kir2.1 trafficking (Kolb et al., 2014).

In subsequent yeast studies, the α-arrestins, a class of selective protein trafficking adaptors (O'Donnell and Schmidt, 2019), were identified as key regulators of Kir2.1 trafficking (Hager et al., 2018). The α-arrestins selectively bind to membrane proteins and recruit a ubiquitin ligase that in turn ubiquitinates membrane proteins, thus altering their protein trafficking fate. Ubiquitination is a signal for endocytosis and intracellular sorting of membrane proteins (Becuwe et al., 2012; Lin et al., 2008; Nikko and Pelham, 2009; O'Donnell et al., 2010; Nikko et al., 2008) and the α-arrestins are a key facet of selective protein trafficking. In yeast, α-arrestins bind to the ubiquitin ligase, Rsp5, the ortholog of which is mammalian Nedd4-2. Select yeast α-arrestins—namely Ldb19/Art1, Aly1/Art6, and Aly2/Art3—promote Kir2.1 trafficking to the PM, increasing Kir2.1 activity, raising intracellular K^+^ and improving growth on K^+^ restrictive medium. In addition, regulators of these α-arrestins, including Rsp5 and the protein phosphatase calcineurin, which is conserved from yeast to man and is highly expressed in the heart and other excitatory cells where Kir2.1 is active, were also identified in the yeast model (Hager et al., 2018). It remains to be determined if these factors control Kir2.1 trafficking in mammalian cells. It should be noted that studies of Kir1.1 (ROMK) using a yeast model system have also been instrumental in defining key trafficking factors for this family of channels using a similar strategy (Mackie et al., 2018).

## 3 The Role of Kir2.1 in Development and Disease

Given the critical role of K^+^ in action potential firing, it is straightforward to understand how Kir2.1 mutants cause heart arrhythmias in ATS. However, how mutations in Kir2.x genes lead to the developmental defects linked to ATS is somewhat less clear. There is an interplay between patterning of the electrical potential across PMs of cells in developing tissues (referred to as the “bioelectrical pattern”) and activation of developmental signaling pathways. The appropriate pattern of membrane potentials at the PM, regulated in part by K^+^ balance, influences cell migration and orientation during embryogenesis (Levin, 2012). Studies in many organisms, including *Drosophila melanogaster, Xenopus laevis*, and mice, define Kir2.1’s role in development as a modulator of PM potential patterning.

### 3.1 *Drosophila melanogaster* dKirI-III in BMP Regulation

The first hints at the molecular details underlying Kir2.1’s role in development came from studies in *Drosophila*, where Kir homologs were identified in the then newly sequenced *Drosophila* genome. This revealed three presumptive Kir channels—dKirI-III, also known as Irk1-3—where dKirI (Irk1) and dKirII (Irk2) have the highest amino acid similarities to mammalian Kir2.x channels (Döring et al., 2002). Flies deficient for dKirII exhibit wing venation defects, which bear analogy to limb development defects in mammals, including incomplete or branched posterior cross veins, incomplete L5 veins and a bifurcation of the L3 and L4 veins, as well as wing bristle transformations. Thus, there is a conserved over-arching role for Kir2.x channels in development spanning the ∼800 million years of evolution separating flies and man (Dahal et al., 2012; Shih et al., 2015). Inhibition of dKirII causes further developmental abnormalities including held-out wings, thickened wing veins, and small/absent wings as phenotypes, like the phenotypes observed in flies with disrupted bone morphogenetic protein (BMP) signaling. In *Drosophila* the homolog of BMP is the decapentaplegic (Dpp) morphogen which is regulated by its receptor Thickveins (Tkv), a BMP type 1 receptor. Consistent with an overlapping function between BMP signaling and dKirs, the severity of these developmental abnormalities in flies is enhanced when dKirII inhibition is coupled to heterozygous loss of function mutations in BMP signaling factors (Dahal et al., 2012). Further studies demonstrate that dKirII channels are required for Dpp secretion in the developing fly wing disk. Expression of dominant negative alleles of dKirII not only dampened Dpp secretion, but also prevented phosphorylation of Mad, a transcription factor needed for gene expression changes in development (Dahal et al., 2017). The Dpp secretion defect in dKirII deficient flies is overcome by augmenting extracellular K^+^, supporting the idea that K^+^ influx depolarizes the membrane to stimulate Dpp release. Inhibition of Kir2.x channels in excitable cell types alters the resting membrane potential and disrupts calcium homeostasis (Hibino et al., 2010). Similarly, dKir controls calcium levels in developing *Drosophila* embryos, where reduced dKirII diminishes cellular calcium, again preventing Dpp release. Together, these findings demonstrate that dKirII controls Dpp morphogen secretion by permitting K^+^ influx, stimulating cellular calcium spikes and membrane depolarization. The mechanism by which cellular K^+^ and calcium trigger Dpp secretion remains unclear, however, this secretion is needed for proper development (Dahal et al., 2017). This model is conserved in mammals where homozygous Kir2.1 knockout mice similarly exhibit impaired BMP signaling (Belus et al., 2018).

### 3.2 *Xenopus laevis*: Kir2.1 in Bioelectrical Patterning From Frogs to Mice?

A firm connection between Kir2.1 and regulation of the bioelectric patterning during development comes from studies in the African clawed frog. Groundbreaking work in this model system was the first to demonstrate that alterations in bioelectrical membrane patterning cause abnormalities in craniofacial structures, making this an important model system (Vandenberg et al., 2011). In *X. laevis* the homolog of Kir2.1 is expressed in the developing face. Over-expression of Kir2.1 or several ATS-causing Kir2.1 mutants, including T75R and R218W mutations that interfere with trafficking of Kir2.1 (Tani et al., 2007), in *X. laevis* gives rise to craniofacial abnormalities of the eyes, jaw, and branchial arches (Adams et al., 2016), much like the phenotypes observed in mice and ATS patients. Membrane voltage measurements in *Xenopus* embryos where ATS-causing Kir2.1 mRNAs were injected into the animal pole identify Kir2.1-linked changes in resting membrane potential as a cause of cranial facial abnormalities (CFAs). This work adds to the literature showing that proper craniofacial development depends on an intricate pattern of resting membrane potentials (referred to as the bioelectric face prepattern) and Kir2.1 activity is needed to establish this pattern (Adams et al., 2016).

Finally, as alluded to earlier, studies in a mouse model of Kir2.1 demonstrate beautiful conservation of the developmental roles for this channel. In mice, Kir2.1 is expressed in early embryonic states (E8.5 and E9) in the midline of the neural tube, anterior neural tissues, somites and frontonasal processes. By later stages of development (E14.5) during fusion of the palatal shelves, Kir2.1 is strongly expressed in the nasal cavity (Adams et al., 2016; Belus et al., 2018). Given this pattern of expression, it is not surprising that in a mouse model where both functional copies of Kir2.1 are lost, mice survive until birth but display underdeveloped maxilla, mandibles, and nasal bones, as well as cleft palate, small palatine processes and vomer bones, and defective digits as newborns (Dahal et al., 2012; Belus et al., 2018). These morphological changes show that Kir2.1 contributes to skeletal development and help establish Kir2.1 as a regulator of bone development. Bone cells, or chondrocytes, can arise from mesenchymal stem cells (MSC) during chondrogenesis, a process driven in part by transforming growth factor beta (TGF-βs) and BMP signaling pathways. Indeed, expression of the ATS-causing ∆314–315 Kir2.1 mutation in MSCs prevents subsequent chondrocyte differentiation (Pini et al., 2018). Furthermore, induced pluripotent stem cells derived from ATS patients have decreased expression of key chondrogenic markers, including SOX9, which is a pro-chondrogenic transcription factor considered the master regulator of chondrocyte development, and RUNX2 and COLL10A1, which are SOX9-regulated targets and themselves critical downstream effectors in bone development (reviewed in Green et al., 2015) (Green et al., 2015). Loss of Kir2.1 or introduction of ATS-causing mutations in Kir2.1 reduced expression of key extracellular matrix proteins, including collagen 1 alpha (COL1A1) and osteocalcin (OCN), needed for calcification (Pini et al., 2018). Thus, when Kir2.1 function is impaired, the BMP signaling pathway in iPSC-MSCs is disrupted, diminishing downstream Smad phosphorylation, which is analogous to the diminished Dpp and phospho-Mad signaling seen in dKir mutants in *Drosophila*. In future studies, it will be interesting to see if the altered bioelectrical patterning defects observed in *Xenopus* embryos defective for Kir channel function similarly drive BMP secretion defects in mice expressing ATS-causing mutations.

### 3.3 Kir2.1 Beyond Bone Development

While considerable work has focused on Kir2.1 in bone development, Kir2.1 also regulates development of myoblasts, the precursors of excitable muscle cells. Kir2.1-induced hyperpolarization of primary human myoblasts triggers the expression of transcription factors such as myogenic and myocyte-enhancer factor 2 (MEF2) (Konig et al., 2004). Myoblast fusion during muscle development requires Kir2.1 activity (Fischer-Lougheed et al., 2001), and myoblast differentiation into multinucleated myotubes is postulated to occur after dephosphorylation of Kir2.1 at the PM. This aspect of Kir2.1 regulation is thought to be associated with channel gating and activity rather than altering Kir2.1 trafficking or synthesis (Hinard et al., 2008). In differentiating myoblasts, Kir2.1 activity controls transcriptional changes downstream of the protein phosphatase calcineurin; inhibition of Kir2.1 decreases activity of NFAT (nuclear factor of activated T-cells), a transcription factor that is dephosphorylated by calcineurin to prevent MEF2 expression (Konig et al., 2006). Interestingly, studies of Kir2.1 channel trafficking in yeast demonstrate that calcineurin dephosphorylates α-arrestin Aly1, a trafficking adaptor that controls Kir2.1 transit to the PM, to impair Kir2.1 trafficking to the PM (Hager et al., 2018). Although an established, cell surface pool of Kir2.1 regulates myoblast differentiation, it remains unclear whether calcineurin-mediated regulation of Kir2.1 trafficking directly affects Kir2.1-dependent myoblast differentiation. It would be exciting in future studies to see if calcineurin stabilizes Kir2.1 at the PM in myoblasts or other cell types to control membrane potential in development.

## 4 Concluding Remarks and Perspectives

A clearer picture of how disease–linked Kir2.1 mutations impinge on Kir2.1 trafficking and its roles in development has come into view due to recent advances in microscopy and exciting discoveries in bioelectrical patterning. Studies of Kir2.1 have expanded our understanding of protein trafficking, defining novel AP-1 interaction motifs, and channel regulation. However, there remain key outstanding questions in the field centered around the mechanism of Kir2.1 endocytosis and how its trafficking compares to that of other related Kir proteins. It will be interesting to see in the future if intracellular sorting pathways, such as recycling of Kirs from endosomes to the Golgi, might influence the surface expression of this critical K^+^ regulator.

## Nomenclature

AP-1: adaptin Protein complex 1
AP-2: adaptin Protein complex 2
BMP: bone morphogenetic protein
CARC: cholesterol recognition amino acid consensus motif
CIE: clathrin-independent endocytosis
CME: clathrin-mediated endocytosis
COL1A1: collagen 1 alpha
CFA: cranial facial abnormality
ER: endoplasmic Reticulum
ESCRT: endosomal sorting complex required for transport
ERAD: eR-associated degradation
Dpp: decapentaplegic
FA: Focal adhesion
GRIF-1: g-aminobutyric acid receptor-interacting factor 1
**GABA**_**A**_: γ-aminobutyric acid type A receptor
GRIP: Golgin-97, RanBP2alpha, Imh1p and p230/golgin-245
MAGUK: Membrane-associated guanylate kinase
MSC: Mesenchymal stem cells
MEF2: Myocyte-enhancer factor 2
OCN: Osteocalcin
PM: Plasma Membrane
**PIP**_**2**_: Phosphatidylinositol 4,5-bisphosphate
PDZ: Post synaptic density, discs large, and zonaula occludens domain
**K** ^**+**^: Potassium
Kir: Potassium Inwardly Rectifying
Retromer: Retrieval of vacuolar-targeted proteins
Tkv: Thickveins
TRAK2: Trafficking kinesin protein 2
TGN: *trans*-Golgi network
TGF-βs: Transforming growth factor beta
